# Disproportionately enlarged subarachnoid space hydrocephalus presence in patients with idiopathic normal pressure hydrocephalus

**DOI:** 10.1186/2045-8118-12-S1-P43

**Published:** 2015-09-18

**Authors:** Tomas Radovnicky, Daniel Adamek, Milous Derner, Martin Sames

**Affiliations:** 1 Department of Neurosurgery, Masaryk Hospital, Usti nad Labem, Czech Republic; 2 Department of Radiology, Masaryk Hospital, Usti nad Labem, Czech Republic

## Introduction

Idiopathic normal pressure hydrocephalus (iNPH) is a treatable disease of an elderly people. It`s known for 50 years, but there are still some controversies, especially in the diagnostic algorithm. Disproportionately enlarged subarachnoid space hydrocephalus (DESH) seems to be important radiological feature of iNPH, but it`s not present in all patients. The aim of our study was to analyse appearance of DESH in our set of patients.

## Methods

We retrospectively analysed 1, 5 T MRI in 27 iNPH patients before surgery and MRI in 24 healthy controls. Evaluation was performed by neurosurgeon and radiologist independently and blindly. We assessed tight high convexity and medial subarachnoid space, dilatation of Sylvian fissure and focal dilatation of sulci. iNPH patients were identified by clinical examination, dilatation of ventricles on MRI defined by Evans` ratio > 0, 30 and positive tap test and/or lumbar infusion test. Patient outcome was measured by iNPH grading scale one year after surgery.

## Results

In the group of 27 iNPH patients, we have found DESH presence in15 cases (55, 6%), all 15 patients were shunt responders. In the group of 12 iNPH patients without DESH 4 of them did not respond to surgery (33, 3%).

Among the 24 healthy controls, we haven`t found any DESH appearance.

## Conclusion

We confirmed, that DESH appearance on MRI is supportive for the iNPH diagnosis, but it should not be used as a single predictor. Patients without DESH are at a higher risk to be shunt non-responders. Indication for shunt surgery should be still based on correlation between the radiological and clinical evaluation and supplementary CSF dynamics tests.

Supported by IGA NT14448-3/2013.

